# Traditional medicine among the community of Gash-Barka region, Eritrea: attitude, societal dependence, and pattern of use

**DOI:** 10.1186/s12906-021-03247-9

**Published:** 2021-02-19

**Authors:** Sirak Tesfamariam, Filmon Tesfai, Lemlem Hussien, Yonatan Ateshim, Dawit Yemane, Mulugeta Russom, Hagos Ahmed, Iyassu Bahta, Solyana Ngusbrhan Kidane, Josephine Namboze, Ossy Muganga Julius Kasilo

**Affiliations:** 1 Pharmacy, Nakfa Hospital, Nakfa, Eritrea; 2 Traditional Medicine Unit, National Medicines and Food Administration, Asmara, Eritrea; 3 Southern Red Sea Zonal Medical Store, Assab, Eritrea; 4 Eritrean Pharmacovigilance Center, National Medicines and Food Administration, Asmara, Eritrea; 5 Population and Social Statistics Division, National Statistics Office, Asmara, Eritrea; 6 National Medicines and Food Administration, Asmara, Eritrea; 7 Health Systems, WHO Country Office for Eritrea, Asmara, Eritrea; 8 WHO Country Office for Eritrea, Asmara, Eritrea; 9 WHO Regional Office for Africa, Health Systems and Services Cluster, Brazzaville, Republic of the Congo

**Keywords:** Traditional medicine, Community, Pattern of use, Attitude, Children, Gash-Barka, Eritrea

## Abstract

**Background:**

Despite the growing conventional healthcare coverage in Eritrea, traditional medicine (TM) remains an essential source of healthcare service to the population. This study, therefore, aims at exploring the attitude, societal dependence, and pattern of TM use of the Gash-Barka community.

**Methods:**

A cross-sectional study was conducted between December 2018 and January 2019 in Gash-Barka region, one of the six regions of Eritrea. Two-stage stratified cluster sampling design was used to provide representative sample of households. The data collected through face-to-face interview using a structured questionnaire was entered twice and analyzed using CSPro7.2 and SPSS 23, respectively. Both descriptive and analytical analyses were performed to test statistical significance.

**Results:**

Of the total 210 participants, 202 completed the interview with a response rate of 96.2%. Almost 97% of the respondents were aware of the general existence of TM. About half of the respondents (47.4%) had visited traditional health practitioners (THPs) at least once in their lifetime. The majority of the respondents claimed their medical condition had been improved (63.2%), were satisfied with the outcome (76.8%), and had not encountered complications (95.2%) due to TM use. Around 40% of the respondents admitted they do not disclose previous TM use to conventional health practitioners. Females are more likely to have had ever visited THPs (AOR = 1.85, CI: 1.01, 3.38) and use TM in the future (AOR = 2.26, CI: 0.92, 5.14) than males. Moreover, those who had visited THPs before (COR = 8.30, CI: 3.25, 21.20) are more likely to use TM as a primary treatment choice and prefer to use TM in the future (AOR = 4.40, CI: 1.97, 9.83) than those who had never visited THPs. About 61% of the total families claimed they had circumcised at least one female child, and 96.8% disclosed they had circumcised at least one male child. Out of which, 89.2% of the circumcisions were done by THPs.

**Conclusion:**

TM is popular and widely relayed upon by Gash-Barka residents with exposure of children to harmful TM practices. Since the reliance of the community on TM is expected to continue, further representative studies are recommended to inform regulatory interventions and integrate TM into the health system.

**Supplementary Information:**

The online version contains supplementary material available at 10.1186/s12906-021-03247-9.

## Background

Traditional medicine (TM) refers to the knowledge, skills and practices based on the theories, beliefs and experiences indigenous to different cultures, whether explicable or not, used in the maintenance of health as well as in the prevention, diagnosis, improvement or treatment of physical and mental illness [1]. It is as old as humanity itself embedded in the beliefs of communities and it has played an important role in managing both communicable and non-communicable diseases for millennia [2].

Globally, Traditional and Complementary Medicine (T&CM) is more readily available and accessible to patients than conventional medicine [3, 4] even though it is higher in developing countries [2]. As per the WHO report, 88% of all member states reported the use of T&CM [1]. In the last decade, there has also been a global surge in popularity and use of T&CM in both developed and developing countries [5–9]. In which, cultural acceptability [2, 5, 10, 11], perceived efficacy [7, 12], affordability [2, 13, 14], accessibility [7], and psychological comfort [5] of TM along with poor access to modern health services [5, 7, 12] and shortage of conventional health professionals [7], are among the main factors impacting the high degree of utilization.

TM usage continues to be widespread across Africa with over 80% of the population relying on it for their primary healthcare needs [1, 2, 6, 10]. TM practice in Eritrea is no exception. Eritrean TM has a long history that goes hundreds of years back and currently comprises of more than 19 identified modalities of practice that fall into three major categories; namely herbal, spiritual, and procedure-based practices. Previous report shows that there were more than 3900 THPs in Eritrea [15]. Despite the existence of a largely subsidized healthcare services nationally, TM is a widespread phenomenon owing to its cultural acceptability, availability, and accessibility [13, 16, 17]. As such, it still remains essential in maintaining health, preventing, and managing/curing acute and chronic illnesses [18].

The National Medicines and Food Administration of the Ministry of Health (MoH), since the establishment of the Traditional Medicine Unit (TMU) in 2012, has been actively working to regulate TM practices and promote its safe use in the country [1]. As part of its commitments in regulating TM, the ministry ratified a national TM policy in 2017 with a main vision of creating a health care system where both the conventional and traditional systems are joined together to improve universal health coverage [17]. Even though TM is considered important and still practiced by the population, the community’s attitude and level of dependence remains unknown [1, 15]. Besides, the safety of TM practices has been of concern. Therefore, the aim of this study is to explore the attitude, societal dependence, and pattern of TM use of the Gash-Barka community and potential influencers. The findings of this study are expected to fill the gap of research dearth in the area and be used to guide regulatory activities of the MoH as far as TM is concerned.

## Methods

### Study design and setting

A cross-sectional study was conducted between December 2018 and January 2019 for a period of 20 days in randomly selected 15 villages of Gash-Barka region. Eritrea, a country in the horn of Africa, is approximately 120,000 km^2^ large with an estimated population of about 3,095,673 [19]. It is divided into six administrative regions, among which is Gash-Barka region. Gash-Barka, rich with diverse customs and cultural beliefs, is the largest region and a home to eight of the nine Eritrean ethnic groups. The region, which covers most of the western part of the country, is a residence for 27.1% of the country’s population [19]. It is subdivided into 14 administrative subzones with a total of 8 towns and 769 villages. According to a recent estimate Gash-Barka has 197,096 number of households [19] and there are 363 conventional health practitioners (CHPs) actively working in the region with certificate, diploma, degree and specialized trainings [20]. On the other side, as per the TMU database, it is estimated that there are 484 THPs practicing diverse modalities of TM [21].

### Study population

Residents of Gash-Barka region were the target population and those who were 18 years old and above and willing to participate in the study were considered eligible. Residents who are deaf or mentally incapable to communicate were excluded from the study.

### Sample size

The sample size was determined by using **n =** $DEFT\ast \frac{z^2 pq}{e^2}$**,** where proportion (p) was taken as 0.5 for no similar study was done before, margin of error (e) ±8%, z statistics for 95% confidence interval (z = 1.96), and design effect of 1.25. The initial sample size calculation yielded 188 households. Then, the sample size was adjusted based on the assumed non-response rate of 10%, and the final sample size was 210 households.

### Sampling design

Two-stage stratified cluster sampling design was used to provide representative sample of households. At the first stage, the 15 villages were selected using probability proportionate to size (PPS) method; where the size measure was the number of households in the villages. List of villages, stratified by sub-zone, with their number of households was used as a sampling frame for the selection of villages.

At the second stage of sampling, systematic random sampling method was employed to randomly select the average 14 households from the available households in each of the selected villages. List of households, obtained from the village administration office, was used as a sampling frame for the selection of the households. Accordingly, the skip pattern between two households was calculated by dividing the total number of households in each village by the required 14 households.

### Data collection tool and approach

The data were collected through face-to-face interview using a structured questionnaire developed by the TMU, and subsequently subjected to face and content validity by the Traditional Medicine Advisory Committee. This committee is a panel of experts whose mandate is to provide the TMU with technical and advisory support. The content validity index (CVI) was calculated at item level, and all items rated as relevant were included to make the I-CVI of the final questionnaire one.

The questionnaire (Additional file 1) consists of three sections designed to capture socio-demographic characteristics, attitude, and pattern of TM use of the respondents. Attitude of the respondents towards TM existence, effectiveness, and support from the community was measured using 13 questions. A raw score of attitude, calculated by three-point Likert scale, was used to find further association, where 5 and 15 were the possible minimum and maximum scores respectively. In the last section, pattern of TM use was assessed through 23 questions designed to measure the extent of TM use, situations that influence TM seeking behavior, outcome of TM practices, future dependence and TM use in children. A pre-test, to check the clarity of the questionnaire, was also conducted among 30 participants resulting in minor modifications on the questionnaire.

After the selection of the households in each village, the head of the family was interviewed - as most of the times he/she determines the family’s health seeking behaviors. If the eligible respondent was not available during the visit, but could be back during the data collection period, a second visit was offered to give him/her the opportunity to participate in the survey. In case of the respondent’s absence during the entire study period for several reasons, replacement by the current person who was taking the health-related decisions was done. When all members of a household were absent on the day of the survey but some members had slept there the previous night, the household was considered as absent. The survey team returned to all absent households before leaving the village, to see if residents were back. If not, this was reported as non-response without replacement. An abandoned household was considered as either a house that had no one living in it for more than 6 months, or the members were unavailable during the entire data collection process. If an abandoned house was encountered during the systematic selection, it was replaced by the next inhabited household.

Two teams each comprising of five experts from the fields of pharmacy, anthropology, botany, pharmacoepidemiology, and organic chemistry participated in the data collection process. A coordinator, two supervisors, and two field leaders were additionally recruited to ensure the quality of the collected data.

### Statistical analysis

All completed questionnaires were entered twice; that is 100% verification was done to eliminate keying errors during entry, using CSPro version 7.2 (Census and Survey Processing System) software. Data cleaning was carried out using CSPro, both during and after data entry. After data entry, data cleaning and analysis were supported by the Statistical Package for Social Science Version 23 (SPSS 23).

Both descriptive and analytical analyses were made, including the production of cross tabulations between variables. Normality was checked for continuous variables using Kolmogorov-Smirnov and Shapiro-Wilk’s test. Chi-square test for independence has been employed to test the statistical significance of the association between attitude and pattern of TM use and different socio-demographic characteristics of the respondents. Bivariate binary logistic regression analysis was implemented to estimate the crude odds ratio of the impact of the socio-demographic characteristics on attitude and pattern of TM use among the respondents. Multivariate analysis was done to identify the significant socio-demographic characteristics of the respondents in determining the attitude and pattern of TM use by controlling the effect of other factors. On the other side, non-parametric tests: Kruskal-Wallis and Mann-Whitney U tests; were carried out to find out associates of not normally distributed variables.

As the sample design was not self-weighted, all analyses were carried out on weighted number of cases. This was based on the probabilities of selection of the villages and households within the selected subzones taking the non-responses into account. Finally, statistical significance test was measured at 5% level of significance.

## Results

### Demographic characteristics of the respondents

Of the total 210 participants recruited, 202 completed the interview yielding a response rate of 96.2%. The majority of the respondents were females (59.2%), aged between 35 and 54 (46.2%), married (79.6%), Muslims (59.8%), and illiterate (54.7%). Subjects of the study were from diverse ethnical backgrounds where the distribution was dominated mainly by Tigre (34.7%) followed by Tigrigna (28.4%), and Kunama (20%). The detailed demographic characteristics of the respondents is presented in Table 1.

**Table 1:** Distribution of the respondents by their background characteristics. Ethnicity: Others* *contain Saho, Bilen, Nara, Afar*

Variables	Number of respondents	Percentage
**Sex**		
Male	82	40.8
Female	120	59.2
**Age** (Median **=** 45, Range = 18 to 90)		
Below 35	42	20.8
35–54	93	46.2
55 and above	67	33.0
**Marital status**		
Single	10	5.0
Married	161	79.6
Divorced	13	6.3
Widowed	18	9.0
**Educational level**		
Illiterate	110	54.7
Elementary	61	30.2
Junior	23	11.2
Secondary	6	3.0
Higher education	2	1.0
**Ethnicity**		
Tigre	70	34.7
Tigrigna	57	28.4
Kunama	40	20.0
Hidareb	18	8.8
Others*	16	8.1
**Religion**		
Christian	80	39.8
Muslim	121	59.8
Animist	1	0.4
**Household size**		
Below 5	67	33.1
5 or above	135	66.9

### Attitude of the community towards TM

Almost 97% of the respondents were aware of the general existence of TM, while the remaining claimed that they have never heard of TM previously. Forty-two percent of the respondents admitted that they had come across individuals who visited health facilities soon after trying to manage their disease condition using TM. The reasons mentioned that led to health facility visit after discontinuing TM usage, according to the respondents’ attitude, were worsened disease condition (88.4%), delayed improvements (77.7%), peer influence (4.7%), and unknown/undisclosed reasons (11.1%). (**Note:** As multiple choices were possible, the total percentage calculated might exceed 100%).

Regarding the preference of healthcare, 81.1% of the respondents disclosed that they prefer conventional medicine over TM, 4% prioritize the opposite, and the remaining 14.9% do not have specific preference and use both traditional and conventional medicines based on the nature of the disease. One-fourth (26.5%) of the respondents believe that the community still supports TM practices and a number of factors were identified concerning the reasons why the community seeks TM. Religious or cultural convictions (44.6%), safety and effectiveness of TM (29.3%), and lack of awareness towards the safety of TM and/or conventional medicine (17.1%) were among the major ones (Table 2).

**Table 2:** Percentage distribution of the respondents’ attitude on why people visit THPs

Reason	Number of respondents	Percentage
Religious or cultural conviction	87	44.6
TM is more effective & safer than conventional medicine	57	29.3
Lack of awareness towards the safety of TM and/or conventional medicine	33	17.1
When the respondents were told that their case could not be treated with conventional medicine	31	15.9
TM is more accessible than conventional medicine	29	14.7
TM is more affordable than conventional medicine	18	9.0
Fear of side effects from conventional medicine	6	3.0
Other reasons	14	7.1
Don’t know	10	5.0

Furthermore, 27% of the respondents believe that there are some diseases that can only be cured using TM; psychiatric illnesses (59.6%) and *Buda* (a form of evil spirit possession) (40.4%) were the two disease conditions reported by the respondents. Nearly six in ten (59.6%) of the respondents supported the discontinuation of TM practice in general.

### Association testing of the community’s attitude towards TM

Raw score of attitude with one-point increment was calculated, a higher score indicating positive attitude towards TM and, 15 and 5-points were the possible maximum and minimum scores, respectively.

As most of the independent variables were not normally distributed, appropriate non-parametric tests; Kruskal-Wallis test for variables with more than two categories and Mann-Whitney U test for variables with two categories, were performed to identify possible difference in the distribution of attitude score among the categories of individual variables (Table 3). From the five variables tested, only “Ever visited a THP” (*p* <  0.0001) was found to have significant difference in the distribution of attitude score across its categories. The result indicated that respondents who had used TM (Median = 11) were found to have significantly higher median attitude score than those who had never used (Median = 8).

**Table 3:** Difference in attitude score of the respondents by demographic characteristics using appropriate non-parametric tests

Variables	Median score	***p-value***
**Age**		0.138
Less than 35 years	10	
35 to 54 years	9	
Greater than 54 years	10	
**Ethnicity**		0.124
Kunama	10	
Tigre	10	
Tigrigna	9	
Others	9	
**Sex**		0.111
Male	9	
Female	9	
**Level of education**		0.568
No formal education	9	
Elementary or higher	9	
**Ever visited a THP**		*< 0.0001*
Yes	11	
No	8	

### Pattern of TM use

Almost half of the respondents (47.4%) had visited THPs at least once in their life time in which 19% of them visited in the past year. According to the sex of the respondents, females (61%) visited more compared to males (39%). Different situations shown in Table 4 were mentioned to have caused TM seeking behaviors.

**Table 4:** Situations when respondents seek treatment from TM

Situations	Frequency	Percentage
To manage rare/certain disease conditions	71	36.6
Every time I get sick	11	5.6
Whenever my case is said untreatable by conventional medicine	7	3.8
Whenever I’m far from the conventional medicine	6	3.2
Never used TM	103	52.6

When respondents were asked about the outcome of their last visit to a THP, 63.2% of them said that the illness/medical condition had improved while 28.6% reported that they had not observed any changes at all. Likewise, more than three-fourth of the respondents were satisfied with the outcome of TM either wholly (48.6%) or partially (28.2%). Most of the respondents (95.2%) stated that they have not encountered any complications as result of TM use (Table 5).

**Table 5:** Impression of respondents on the outcome and practice of TM

Questions		Percentage
Outcome of the recent TM used	Improved	63.2
	Exacerbated	1.1
	No change	28.6
	Cannot recall	7.2
Complications encountered after the use of TM	Yes	4.8
	No	95.2
Satisfaction level of the TM service received	Wholly satisfied	48.6
	Partially satisfied	28.2
	Not satisfied	23.3
Plan to use TM in the future	Yes	20.3
	No	69.7
Encouraging others to use TM	Yes	19.4
	No	70.6
Willingness to disclose use of TM to CHPs	Yes	60.9
	No	39.1

Results for future usage of TM shows that one-fifth (20.3%) of the respondents have plan to visit THPs in the future, and 19.4% admitted that they encourage people to use TM too because they believe that TM is effective or for other undisclosed reasons. Additionally, almost two-third (60.9%) of the respondents reported that they were willing to disclose any TM taken by them or given to their children to CHPs. On the contrary, the remaining 39.1% admitted that they do not disclose previous TM use because they did not see the importance of doing so. Fear of criticism by CHPs was also another reason for not disclosing information. On choosing a THP, the community base their decision on the popularity of the THP (47.3%), nature of their illness (42.0%), accessibility of the healer (18.7%), concordance between their and the healers’ cultural identity or religious belief (10.2%), and affordability of the services (8.1%).

### Influencers of the community’s pattern of TM use

The potential associates of community’s pattern of TM use identified were age, sex, marital status, educational level, ethnicity, religion, and previous THP visit. The magnitude and direction of relationship of all these variables with the pattern of TM use was assessed using bivariate logistic regression. Variables that were found to be significant along with their odds ratios are displayed in Table 6.

**Table 6:** Bivariate and multivariate analysis on the association between respondents’ demographic characteristics and their pattern of TM use. Ethnicity: Others*: *Hidareb*, *Saho, Bilen, Nara, Afar*

Variables	Bivariate analysis			Multivariate analysis		
	COR	95% CI	***p***-value	AOR	95% CI	***p***-value
***Preference of TM as a primary healthcare***						
**Ever visited THP**						
Yes	8.30	(3.25, 21.20)	< *0.0001*			
No	*Ref*					
***Ever visited THPs***						
**Sex**						
Female	1.78	(0.99, 3.17)	0.052	1.85	(1.01, 3.38)	*0.046*
Male	*Ref*					
**Ethnicity**						
Tigre	0.85	(0.42, 1.73)	0.655	0.97	(0.47, 2.01)	*0.934*
Kunama	2.05	(0.88, 4.78)	0.097	2.30	(0.97, 5.46)	*0.600*
Others*	*0.60*	(0.25, 1.47)	0.263	0.64	(0.26, 1.59)	0.338
Tigrigna	*Ref*					
***Future use of TM***						
**Sex**						
Female	2.60	(1.17, 5.75)	0.018	2.26	(0.92, 5.14)	< *0.0001*
Male	*Ref*					
**Ever visited THP**						
Yes	4.73	(2.13, 10.50)	< *0.0001*	4.40	(1.97, 9.83)	< *0.0001*
No	*Ref*					
***Use of TM to induce diarrhea and/or vomiting for “cleansing purposes”***						
**Level of Education**						
No formal education	2.48	(1.11, 5.54)	0.026			
Elementary or higher	*Ref*					
***Willingness to disclose use of TM to CHPs***						
**Ethnicity**						
Tigrigna	9.03	(2.98, 27.36)	< *0.0001*	8.74	(2.85, 26.75)	< *0.0001*
Tigre	1.81	(0.59, 5.52)	0.300	1.72	(0.56, 5.31)	0.345
Kunama	7.02	(2.20, 22.41)	0.001	5.97	(1.84, 19.37)	0.003
Others*	*Ref*					

“Ever visited to a THP” was found to be a factor that most influenced the successive practice of the respondents on TM. Those who had visited THPs before were eight times (COR = 8.30, CI: 3.25, 21.20) more likely to use TM as a primary treatment of choice compared to those who had never visited THPs. Similarly, those who had visited THPs have four times more preference to use TM in the future (AOR = 4.40, CI: 1.97, 9.83) than those who had never visited.

Revealing the influence of sex, females were twice (AOR = 1.85, CI: 1.01, 3.38) more likely to had ever visited THPs before than males, and at the same time twice (AOR = 2.26, CI: 0.92, 5.14) more likely to use TM in the future. Respondents with no formal education were 2.5 times (COR = 2.484, CI: 1.11, 5.54) more likely to practice TM on their children for cleansing purposes of the gastro-intestinal tract (GIT) by inducing diarrhea and /or vomiting than those who have formal education. Additionally, Tigrigna (AOR = 8.74, CI: 2.85, 26.75) and Kunama (AOR = 5.97, CI: 1.84, 19.37) ethnic groups were more likely to resist disclosing use of TM to CHPs than the other category of ethnic groups.

### Use of TM in children under the age of 15 years

Most of the participants (78.9%) admitted that TM can impose potential risks on children’s health while 2.4% believed that TM is the safest. About one-fifth (19.2%) of the respondents had sought protection from evil eye either for themselves or for their children, and 17.7% of them used TM to induce diarrhea and/or vomiting for their children as a way of cleansing the GIT.

When respondents were asked what they would do if their child’s medical condition worsened while taking conventional medication, most of them (90%) stated that they would get back to a health facility to seek further treatment. On the other hand, 5% reported that they would visit THPs, and 5% admitted they would either stop the medication or continue following up the full regimen.

In this study, 60.7% of the total families claimed that they had circumcised at least one female child, and 96.8% disclosed that they had circumcised at least one male child. Out of which, 89.2% of the circumcisions were done by THPs. Meanwhile, 63.3% of the study’s families claimed that at least one of their children had undertaken uvulectomy and 94.1% of the procedures were performed by THPs.

## Discussion

Most of the respondents were aware of the presence of TM in their community indicating that it is a common practice in Gash Barka region. Even though the majority of the respondents disclosed that they prefer conventional medicine to TM, this preference is not consistently reflected in their actual practices. In this study, the extent of TM use, at least once in a lifetime, was approximately 50% similar with findings reported from developed countries [2]. The result was lower comparing to the WHO estimate 80% African population reliance [1]. Meanwhile, studies conducted in Southeast Nigerian [22] and Ethiopia [10, 23, 24] also reported a higher prevalence of TM use ranging from 73.8 to 94.2%. Moreover, the conventional healthcare system of the Gash Barka region includes 14 physicians (1 physician to 59,941 inhabitants) compared to 484 THPs (1 THP per 1734 inhabitants). High per capita distribution of THPs could be one reason for the widespread utilization of TM. This indicates that TM is offering an extensive support to the Eritrean healthcare system to attain the purpose of universal health coverage.

The higher practice of TM by females compared to males in this study is consistent with findings conducted in Saudi Arabia [25] and Europe [26], whereas the opposite was reported in one Nigerian study [22]. Higher rate of TM by females in this result could be attributed to the higher likelihood of females’ involvement in childcare than males. As per the impression of the authors, in Gash-Barka region, most of the males are farmers and merchants, their job mandates to spend a lot of time away from the family, thus giving space to females taking health related decision of the family, could be an additional factor.

There are a lot of motivating reasons that push the community to seek treatment from TM [11, 13, 22]. In this study, compatibility among the traditional treatments, religious, and cultural beliefs was found to be the main contributors. Additionally, the population’s attitude on the effectiveness of TM in curing/managing certain/rare disease conditions was another motivating factor [2, 4, 13, 23]. For instance, as per the current study respondents believe; psychiatric illness and *Buda* are disease conditions that can only be cured by THPs. In such cases, the community will only use alternative ways indicating dependence on TM will continue and TM will remain an important component of public healthcare.

Further results were found to support the notion that the community will continue seeking medical treatment from TM. Two-thirds of the present study participants admitted that their medical conditions had been improved; three-fourth satisfied with the outcome, and most had reported that they did not encounter treatment complications after the use of TM similar to studies done in southeast Nigerian [22] and West Ethiopia [23]. At this point, it is important to discuss the possible influencers of the result on the reported treatment complications. First, the community might not recognize adverse events due to lack of awareness and consider the treatment complications as part of either the diseases or the TM effect. Secondly, traditional birth attendants dominated distribution of the THPs of the region [21] and the natural phenomenon of child labor associated with low harmful effect on the mother, unless the parturition is complicated, could have affected the low report of adverse events.

Revealing the association results, study participants who had visited THPs before were found to have significant positive attitude, preference of TM as a primary treatment of choice, and were more likely to use TM in the future compared to those who had never visited THPs. Perceived effectiveness of TM [5, 10, 13], satisfaction level [12], and minimum treatment complications encountered could have possibly motivated the respondents who had used TM to further visit THPs. Rurality [27], educational level [22, 26], marital status [22], and income [22] were found to influence subsequent T&CM practices in several studies.

Reluctance in disclosing use of TM to CHPs is common [25, 28, 29]. In this survey, around 40% of the study participants admitted that they do not disclose previous TM use or visit to CHPs, for several reasons. The main reasons reported- fear of physician’s criticism or failure of healthcare professionals to ask about previous TM use, are worth discussing. As respondents might not think revealing previous use of TM is important, thereby possibly interfering with modern medicine diagnosis, CHPs should request in the first place without expecting from patients at all [30]. Herbal medicines are often perceived by patients and consumers as being natural, hence safe and with low or no side effects [31]. Some studies have, however, highlighted that herbal medicines can interact and may result in adverse reactions if co-administered with conventional medicines [22, 31, 32]. Since, most patients are often on prescription or non-prescription medicines, it is important that CHPs develop an information seeking habit on previous use of TM for a better outcome of medical treatment [29]. Besides, Tigrigna and Kunama ethnic groups were found to be reticent in disclosing TM use. The reasons behind such behavior could not be identified due to lack of qualitative data; further study is, thus, warranted.

The results indicated that children had been subjected to harmful practices. Reported uvulectomy and circumcision of both male and female children under the age of 15 by THPs was common. Since the denominator was not determined, it is worth noting that the results do not portray the actual prevalence. The risk is heightened as these practices are often associated with poor hygienic procedure and use of unsterilized tools. Use of herbs for GIT cleansing purposes in children, was another widely reported practice. Parents with no formal education were more likely to administer herbal materials for such purposes, indicating that educational level of respondents was an influencing factor. Since safety continues to be a major issue with the use of TM practices in general, it is imperative for regulatory authorities to exercise appropriate measures to ensure the safety of the community.

### Limitations

There are at least three limitations: firstly, the sample size was adequate for the studied region only and therefore, the results cannot be generalized to the whole country. Secondly, the percentage of circumcision reported might not be a good proxy as we did not perform any physical examination to determine whether a child was circumcised or not. Finally, the use of different translators and recall bias on the previous history of TM use could have affected the accuracy of the data.

## Conclusions

TM is popular and widely practiced among the residents of Gash-Barka region. Previous visit to a THP and sex of the respondent were found to significantly influence the subsequent use of TM. Additionally, religious and cultural inclinations, high satisfaction level, and positive outcome of TM use are key explanatory factors as to why people still continue to use TM or even prefer it to conventional medicine. Since the reliance of the community on TM is expected to continue, the MoH should promote studies to assess the benefits and risks of TM practices, establish their safety, and understand THPs’ and CHPs’ attitude towards integration to successfully integrate TM into the health system and attain universal health coverage. Apart from that there was lesser tendency on the side of the community in disclosing information regarding previous TM use to CHPs. There is a need for raising awareness of the community and CHPs on the importance of disclosing such information. Circumcision, uvulectomy, and use of herbs with no established safety profile are among the widely practiced TM activities on children. The risk to which children are being exposed is high and this calls for an immediate attention from concerned bodies.

## Supplementary Information

**Additional file 1.** Questionnaire used for the assessment of attitude, and pattern of traditional medicine use in the Gash-Barka community.

## Data Availability

The complete dataset used in statistical analysis and supporting the conclusion of this article could be retrieved from the corresponding author upon a reasonable request.

## Abbreviations

AOR: Adjusted Odds Ration
CHP: Conventional Health Practitioner
CI: Confidence Interval
COR: Crude Odds Ratio
CSPro: Census and Survey Processing System
CVI: Content Validity Index
MoH: Ministry of Health
PPS: Probability Proportionate to Size
SPSS: Statistical Package for Social Sciences
T&CM: Traditional and Complementary Medicine
TM: Traditional Medicine
THP: Traditional Health Practitioner
TMU: Traditional Medicine Unit
WHO: World Health Organization
