# Supplementary material for: Traditional medicine among the community of Gash-Barka region, Eritrea: attitude, societal dependence, and pattern of use
Source: BMC Complement Med Ther. 2021 Feb 19;21:68. doi: 10.1186/s12906-021-03247-9 (PMC7893741; doi:10.1186/s12906-021-03247-9)
Supplement: Supplementary file 1 — Additional file 1. Questionnaire used for the assessment of attitude, and pattern of traditional medicine use in the Gash-Barka community. [file 12906_2021_3247_MOESM1_ESM.pdf]

THE STATE OF ERITREA

MINISTRY OF HEALTH

Assessment of Attitude and Pattern of Traditional Medicine Use in the Gash-Barka Community, 2018

ALL INFORMATION COLLECTED IS CONFIDENTIAL AND IS ONLY FOR STATISTICAL USE

IDENTIFICATION PARTICULARS	
Zone..	☐
Sub-Zone	☐☐
Village/Town Name [Urban=1, Rural =2]________________________________	☐
Administration..	☐☐☐
Cluster No...	☐☐☐
Household Number..	☐☐☐

Date of interview _________/________/________

Result Code ☐ 1 = Completed 2 = Partly completed 3 = Refusal converted
4 = Refusal 5 = Unable to respond 6 = Other

Questionnaire Language ☐☐ Native Language of Respondent ☐☐ Language of Interview ☐☐

01= Afar	02= Bilen	03= Bidawiet	04= Kunama	05= Nara
06= Arabic	07= Saho	08= Tigre	09= Tigrigna	10= Other

Translator used? ☐ 1= Not at all 2= Sometimes 3= All the time

**Interviewer's Name & Code**	**Supervisor's Name & Code**	**Field Editor's Name & Code**	**Keyed by**
________________ ☐☐	________________ ☐☐	________________ ☐☐	☐☐

Demographic Information

No.	Indicators	Coding Categories	Skip
101	Age of respondent in completed years	Age ☐☐	
102	Year of birth	Year ☐☐☐☐	
103	Sex	Male .... 1<br>Female 2	
104	Ethnicity	Tigrina .... 1<br>Saho .... 2<br>Kunama .... 3<br>Bilen .... 4<br>Rashida .... 5<br>Tigre .... 6<br>Afar .... 7<br>Hidareb .... 8<br>Nara .... 9<br>Other *(please specify)* ___________ 10	
105	Religion	Christian .... 1<br>Muslim .... 2<br>Animist .... 3<br>Other *(please specify)* ___________ 4	
106	Languages<br>*(you are fluent in)*	Tigrina .... A<br>Saho .... B<br>Kunama .... C<br>Bilen .... D<br>Arabic .... E<br>Tigre .... F<br>Afar .... G<br>Bidawiet .... H<br>Nara .... I<br>Other *(please specify)* ___________ J	
107	Level of education	None .... 1<br>Primary school .... 2<br>Middle school .... 3<br>Secondary school .... 4<br>Higher education 5	
108	Marital status	Married .... 1<br>Separated/Divorced .... 2<br>Widowed .... 3<br>Not married, but living together .... 4<br>Single 5	
109	Main occupation	___________	
110	Family size	☐☐	
111	Family income per month	___________	

KAP of Traditional Medicine

No.	Indicators	Coding Categories	Skip
201	Have you ever heard about Traditional Medicine (TM)?	Yes .... 1<br>No 2	(No) → **END OF INTERVIEW**
202	What is your primary source of healthcare services?	Traditional Medicine .... 1<br>Conventional Medicine .... 2<br>Both 3	
203	Is there a Traditional Medicine Practitioners (TMPs) in your area?	Yes .... 1<br>No .... 2<br>Don't know 3	(No / Don't know) → **205**
203A	Give names of TMP(s) *(if willing):*	____________________<br>____________________<br>____________________	
204	Which ones do you know?<br>***Multiple answers are possible***	Herbalists .... A<br>Bone-setters .... B<br>Exorcists .... C<br>Witch doctors .... D<br>Circumcisers .... E<br>Blood letter .... F<br>Massager .... G<br>TBA .... H<br>Other *(please specify)* __________ X	
205	Do you think TMPs are popular in your area?	Yes .... 1<br>No 2	
206	TM is cheap and accessible.	Strongly Agree .... 1<br>Agree .... 2<br>Neutral .... 3<br>Disagree .... 4<br>Strongly disagree 5	
207	Have you come across patients who visited modern health care service soon after visiting TMP?	Yes .... 1<br>No 2	(No) → **209**
208	Why he/she did so?	No improvements .... A<br>Side effect of the treatment .... B<br>Peer influence .... C<br>Don't know .... D<br>Other *(please specify)* __________ X	
209	What treatment do you consider to be the safest in treating your sick child?	Traditional Medicine .... 1<br>Conventional Medicine .... 2<br>Both 3	
210	Do you think there are potential risks that TM imposes on a child's health?	Yes .... 1<br>No .... 2<br>Depends .... 3<br>Don't know 4	

No.	Indicators	Coding Categories	Skip
211	Do you agree with your community's support of TM practices?	Strongly Agree ....1<br>Agree ....2<br>Neutral ....3<br>Disagree ....4<br>Strongly Disagree 5	
212	How often do you visit a TMP (if you have before)?<br><br>***Multiple answers are possible***	Every time I get sick ....A<br>Whenever my case is said untreatable by conventional medicine ....B<br>Whenever I'm far from the conventional medicine ....C<br>Rarely ....D<br>Never .... E →	**217**
213	Have you visited a TMP in this year?	Yes.... 1<br>No ....2 →	**214**
213A	How many times have you been?	☐☐	
214	What was the outcome of your treatment, in your most recent visit, to a TMP?	Improved....1<br>Exacerbated ....2<br>No change ....3<br>Cannot recall....4	
215	Have you encountered treatment complications or adverse events from a therapy you received from traditional healer?	Yes.... 1<br>No 2	
215A	Specify (if **yes**):	________________<br>________________	
216	How satisfied are you with the treatment you receive from the traditional healers you visited?	Wholly satisfied.... 1<br>Partially satisfied .... 2<br>Not satisfied.... 3	
217	Do you plan to use TM in the future?	Yes.... 1<br>No 2	
217A	If **No**, reason(s) why?	________________<br>________________	
218	Do you encourage others to use TM?	Yes.... 1<br>No 2	
218A	If **Yes**, specify why?	________________<br>________________	
219	Have you sought protection for (yourself/children) from evil eye via TM?	Yes.... 1<br>No 2	
220	Do you use TM for your child to induce diarrhea and/or vomiting for "cleansing purposes"?	Yes.... 1<br>No 2	
220A	Specify the kind (if **Yes**):	________________<br>________________<br>________________	

No.	Indicators	Coding Categories	Skip
221	If your child became sicker while on conventional medicine, what would you do?<br>***Multiple answers are possible***	Stop the medicine ....A<br>Go back to the clinic ....B<br>Continue until the medication is finished ....C<br>Go to the traditional healer ....D<br>Other ________________________________X<br>____	
222	Do you disclose any alternative treatments that you took (or gave your child) to your doctor or other health professional during your visit to health facility?	Yes... 1<br>No 2	
222A	If **No**, reason why?	____<br>____	
223	If you were asked would you talk to them about it?	Yes... 1<br>No 2	
224	Do you believe that traditional healers are knowledgeable of their practices?	Strongly Agree ....1<br>Agree ....2<br>Neutral ....3<br>Disagree ....4<br>Strongly Disagree 5	
225	Do you use traditional remedies at home?	Yes... 1<br>No 2	→ **230**
226	What were the main sources of supply for your traditional remedies/products?	From Practitioners ....A<br>Neighbours ....B<br>Relatives ....C<br>Friends ....D<br>Acquired it myself .... E<br>Other ________________________________X<br>____	
227	What formulations of TM were taken?	Ingestible/powder form ....A<br>Drinkable/liquid form ....B<br>Ointment ....C<br>Other ________________________________X<br>____	
228	Can you give some examples along with the disease conditions you use it for?	____<br>____<br>____<br>____<br>____	
229	Do you think there are diseases that cannot be cured by modern medicine but can be by TM?	Yes... 1<br>No ....2<br>Don’t know..3	
229A	Mention some of the diseases along with their cures:	____<br>____<br>____<br>____	

No.	Indicators	Coding Categories	Skip
230	Do you prefer to receive treatments from TM to conventional medicine?	Always ....1<br>Sometimes ....2<br>Only for some conditions ....3<br>Never 4	
231	What are your preferences in picking a particular TMP over another?	Nature of my illness ....A<br>Cost ....B<br>Accessibility (near to my area) ....C<br>Religious/cultural ....D<br>Popularity of the TMP .... E<br>Other ________________X<br>________________	
232	Why do you think people visit TMP?<br>***Multiple answers are possible***	When they are told that their case cannot be treated with conventional medicine .... A<br>They believe that TM is more effective & safer than conventional medicine .... B<br>TM is more affordable than conventional medicine .... C<br>TM is more accessible than conventional medicine .... D<br>Religious or cultural conviction .... E<br>Fear of side effects from conventional medicine .... F<br>Other ________________ X<br>________________	
233	Do have children under 15 years in the household?	Yes.................... 1<br>No....................2	→ **238** (No)
234	Are the children (in the household) circumcised?<br>a) Male(s)<br>b) Female(s)	**Yes** **No**<br>a) Male(s) 1....................2<br>b) Female(s) 1 2	***If* No *for both skip to* 235**
235	Where the children (in the household) have undertaken such procedure?	Modern health facility ....1<br>Traditional circumciser ....2<br>Other ________________X	
236	Have you undertaken uvulectomy in your childhood?	Yes.................... 1<br>No....................2	→ **238** (No)
237	Where have you undertaken such procedure?	Modern health facility ....1<br>Traditional uvulectomist ....2<br>Other ________________X<br>________________	
238	Do you believe TM practices should be stopped?	Strongly Agree ....1<br>Agree ....2<br>Neutral ....3<br>Disagree ....4<br>Strongly Disagree 5	

Thank you for completing the interview!
